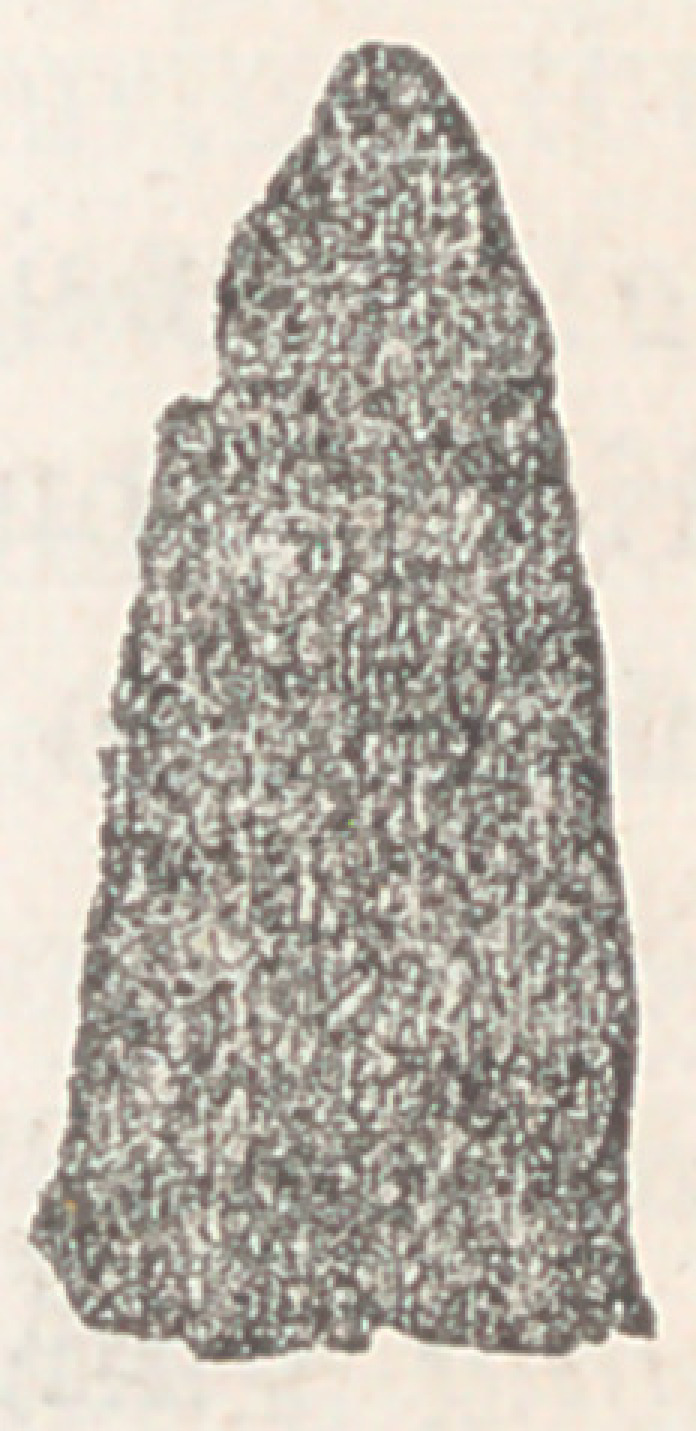# A Fragment of Knife-Blade Lodged in the Chest for Twelve Years, and Finally Coughed up

**Published:** 1870-07

**Authors:** J. F. Snyder

**Affiliations:** Virginia, Cass Co., Ill.


### CHICAGO MEDICAL EXAMINER.

N. S. DAVIS, M.D., Editor.

VOL. XI. JULY, 1870. NO. 7.

### Original Contributions.

### ARTICLE XVII.

### A FRAGMENT OF KNIFE-BLADE LODGED IN THE CHEST FOR TWELVE YEARS, AND FINALLY COUGHED UP.

Case Reported by J. F. SNYDER, M.D., of Virginia, Cass Co., Ill.

[To the Morgan Co. Medical Society, June 9th, 1870.]

Of the remarkable records in medical literature of extensive lacerations of the living tissue, hedged in and restored by the curative process of Nature—and in spite, sometimes, of meddlesome, surgical interference—there are none more marvelous, and but few which better illustrate the peculiar *vis conservatrix* inherent to the organized system, than the many wonderful recoveries from grave injuries of the thoracic organs.

The dissecting room has revealed great cicatrices of the lungs, where cavities, left by broken down tubercles, have been arrested in their destructive progress and healed by this subtile life-power.

Persons have lived for years, in comparative good health, with pieces of cloth, paper, cartridges, fragments of steel and bullets encysted in the parenchyma of the lung—that vital organ which, in sleep or wakefulness, never rests for a minute, from birth until death.

Dr. Druitt relates the case of a soldier, who had "for years a leaden ball rolling about loose in the cavity of the pleura, without occasioning pain or inconvenience."

Mr. J. Bell tells us of "a surgeon who was dabbling in the thorax, with a piece of caustic, which fell directly into the cavity of the chest, where it caused very large suppurations, and yet the patient was saved—he recovered, in spite of the surgeon."

Gen. James Shields, formerly representative of this State, in the U. S. Senate, was shot, during the Mexican war, with a copper ball, weighing an ounce, which passed *through* his chest, penetrating the right lung, a little below its centre; yet his recovery was speedy and complete.

The following strange case of tolerance of a foreign body in the thorax, and its spontaneous removal, came under my observation, not long since; and I have deemed it worthy of record, as a curiosity of surgical pathology.

James Thompson, sixty years of age, stout and robust, usually, of active habits, suddenly commenced declining in health, without apparent cause. When I was consulted, he had been, as he expressed it, "under the weather for five or six weeks." His symptoms were a troublesome, dry cough, furred tongue, loss of appetite, emaciation, hectic night-sweats, and pain in the right side. Previous to the initiation of this train of symptoms, which he attributed to "catching cold," he had always enjoyed excellent health, "excepting," as he said, "occasional twinges of rheumatism, for the last dozen years, under the right shoulder-blade," whenever he exerted himself at any kind of manual labor.

The chest examined, revealed a portion of the right lung, two or three inches in diameter, just below the nipple, entirely impervious to air, and all the organ below that, very dull, on percussion. The left lung was evidently healthy, though overtaxed by its vicarous labor.

The *diagnosis* suggested was *circumscribed pneumonia*, originating, perhaps, in the increasing size and consequent pressure of some isolated tubercular mass. (I will here state that the patient's wife died a few years before of *phthisis*, and it is possible I was influenced in my conclusion by a vague idea of the contagious theory of that disease.)

The *treatment* ordered consisted of stimulating expectorants, mineral acids, and counter-irritants. For four weeks more the case continued without change, save a gradual aggravation of all the symptoms, increased dyspnœa, and free expectoration, when one day, in a hard paroxysm of coughing, the patient threw up, from the right bronchia, an ounce or two of pus and a hard substance, which attracted his attention, by the force with which it struck the floor. On examining the substance, it proved to be the *point of a knife-blade*, an inch in length, half an inch in width, and weighing half a drachm. The fragment of steel was much corroded and pitted by oxydation, as will be seen by the subjoined photograph of actual size.

The patient now remembered a circumstance he had entirely forgotten—that *twelve years* before this, in a street fight, at Beardstown, in which himself and several others had been engaged, he had been "stabbed in the back, about the lower point of the shoulded-blade," but as the wound gave him no pain and soon healed, he had no suspicion that any part of the blade had remained embedded in his body. The true pathology of the case was now manifest, and the patient rapidly recovered his health.

The points of interest in this case are,

1st. That such a foreign body should have so long remained fastened in the bone (either the scapula or rib) without causing necrosis or other disturbance, save occasional slight pains, attributed by the patient to rheumatism.

2dly. That, supposing the knife had passed *through* the bone at once, or had penetrated the thoracic cavity, between the ribs, it failed at the time to excite severe inflammation and produce exhaustive suppuration. And

3dly. That, having penetrated the substance of the lung, into the bronchia, by adhesive inflammation and suppuration, it should have been so easily expelled through the *thyreo-arytenoid ligaments* and the *glottis*.

## Figures and Tables

**Figure f1:**